# Experimental Therapy of Ovarian Cancer with Synthetic Makaluvamine Analog: *In Vitro* and *In Vivo* Anticancer Activity and Molecular Mechanisms of Action

**DOI:** 10.1371/journal.pone.0020729

**Published:** 2011-06-06

**Authors:** Tao Chen, Yi Xu, He Guo, Yanling Liu, Pingting Hu, Xinying Yang, Xiaoguang Li, Shichao Ge, Sadanandan E. Velu, Dwayaja H. Nadkarni, Wei Wang, Ruiwen Zhang, Hui Wang

**Affiliations:** 1 Key Laboratory of Nutrition and Metabolism, Institute for Nutritional Sciences, Shanghai Institutes for Biological Sciences, Chinese Academy of Sciences, Graduate School of the Chinese Academy of Sciences, Shanghai, People's Republic of China; 2 Department of Chemistry, University of Alabama at Birmingham, Birmingham, Alabama, United States of America; 3 Department of Pharmaceutical Sciences, School of Pharmacy, Texas Tech University Health Sciences Center, Amarillo, Texas, United States of America; Vanderbilt University Medical Center, United States of America

## Abstract

The present study was designed to determine the biological effects of novel marine alkaloid analog 7-(4-fluorobenzylamino)-1,3,4,8-tetrahydropyrrolo[4,3,2-de]quinolin-8(1H)-one (FBA-TPQ) on human ovarian cancer cells for its anti-tumor potential and the underlying mechanisms as a novel chemotherapeutic agent. Human ovarian cancer cells (A2780 and OVCAR-3), and Immortalized non-tumorigenic human Ovarian Surface Epithelial cells (IOSE-144), were exposed to FBA-TPQ for initial cytotoxicity evaluation (via MTS assay kit, Promega). The detailed *in-vitro* (cell level) and *in-vivo* (animal model) studies on the antitumor effects and possible underlying mechanisms of action of the compounds were then performed. FBA-TPQ exerted potent cytotoxicity against human ovarian cancer A2780 and OVCAR-3 cells as an effective inhibitor of cell growth and proliferation, while exerting lesser effects on non-tumorigenic IOSE-144 cells. Further study in the more sensitive OVCAR-3 cell line showed that it could potently induce cell apoptosis (Annexin V-FITC assay), G2/M cell cycle arrest (PI staining analysis) and also dose-dependently inhibit OVCAR-3 xenograft tumors' growth on female athymic nude mice (BALB/c, nu/nu). Mechanistic studies (both *in vitro* and *in vivo*) revealed that FBA-TPQ might exert its activity through Reactive Oxygen Species (ROS)-associated activation of the death receptor, p53-MDM2, and PI3K-Akt pathways in OVCAR-3 cells, which is in accordance with *in vitro* microarray (Human genome microarrays, Agilent) data analysis (GEO accession number: GSE25317). In conclusion, FBA-TPQ exhibits significant anticancer activity against ovarian cancer cells, with minimal toxicity to non-tumorigenic human IOSE-144 cells, indicating that it may be a potential therapeutic agent for ovarian cancer.

## Introduction

Ovarian cancer, accounting for about 5 percent of women's cancers death, is now the fifth common cancer in the U.S women [1]. About 70% of all ovarian cancer patients are diagnosed at the more advanced stages of the disease, leading to a poor prognosis [2], [3]. Over the past two decades, although the 5-year survival rate for ovarian cancer patients has been substantially improved owing to the application of successful surgery and the development or optimization of tumoricidal drugs, the survival still remains low, at approximately 30% [2]–[4]. Thus, there is an urgent need to develop novel therapeutic agents that can be used to treat the disease.

Marine organisms are a rich source of potential lead compounds with unique pharmacological properties [5], [6]. Numerous marine natural products with novel molecular structures and diverse biological functions have been reported during the past decades [7]. Makaluvamines, a class of marine pyrroloiminoquinone alkaloids isolated from sponges of the genera *Zyzzya*, have been reported to have potent *in vitro* and *in vivo* cytotoxicity against several human cancer cell lines, and this activity was attributed to their activity as topoisomerase II inhibitors [8]–[14]. Several other makaluvamines have been shown to cause direct DNA damage leading to anti-tumor effects [6], [15].

We have synthesized more than 40 novel makaluvamine analogues and systematically evaluated their anticancer activities in breast cancer cell lines. Our data showed that the potent makaluvamine analog, FBA-TPQ, could inhibit tumor growth through activation of tumor suppressors, inhibition of oncogenes, and activation of the DNA damage response [6], [16]. In the present report, we expanded our study to evaluate the anti-tumor activities of FBA-TPQ against ovarian cancer cells and tried to further elucidate the possible mechanisms of action.

## Results

**Figure 1:**
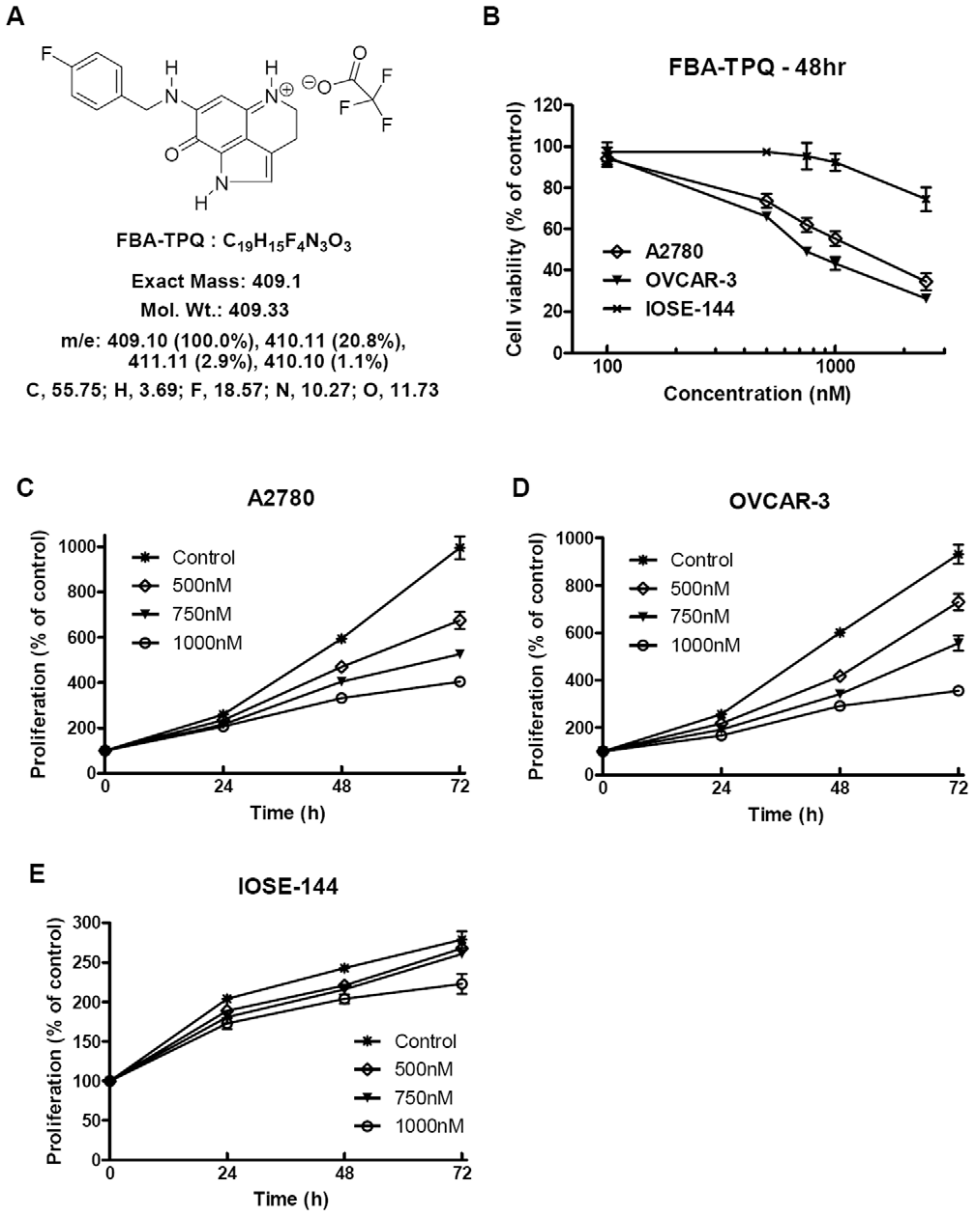
FBA-TPQ preferentially decreases cell viability and growth of A2780 and OVCAR-3 cells but not normal IOSE-144 cells. A, Chemical structure and molecular formula of FBA-TPQ; B, Cell viability (MTS) assay of human ovarian carcinoma cells (A2780 and OVCAR-3) and nontumorigenic OSE cells (IOSE144) after a 48 h incubation with FBA-TPQ; C&D&E, Cell growth inhibition after 0, 24, 48 or 72 h exposure of A2780 (C), OVCAR-3 (D) and IOSE-144 (E) cells to FBA-TPQ(at concentrations of 0, 0.5, 0.75 and 1.0 µM). All values are representative of at least three independent experiments with similar results, and are presented as the percentage of cell growth inhibition, where vehicle-treated cells were regarded as 100% viable (0% growth inhibition).

The *in vitro* cytotoxicity of FBA-TPQ to A2780 and OVCAR-3 cells were first assessed using the MTS assay (Promega). A2780 and OVCAR-3 and human ovarian IOSE144 (Immortalized non-tumorigenic Ovarian Surface Epithelial cells) cells were exposed to FBA-TPQ (see Fig. 1A) at various concentrations for 48 hrs. The viability of the A2780 and OVCAR-3 cells were significantly decreased, with IC_50_ of 1780 nM (A2780) and 980 nM (OVCAR-3) (Fig. 1B). Exposure to FBA-TPQ led to dose-dependent effects on cell survival, inhibiting A2780 viability by 25.2%, 45.7%, and 65.8% while inhibiting OVCAR-3 cells viability by 33.9%, 56.7%, and 73.7%, respectively, for the 0.5, 1.0 and 2.5 µM concentrations, respectively, (Fig. 1, p<0.05). On the other hand, the IOSE144 cells were much less sensitive to FBA-TPQ treatment, with an IC_50_ over 10 µM (Fig. 1B, p<0.01). Exposure to FBA-TPQ decreased the viability of IOSE-144 cells by 2.7%, 7.7%, and 25.6%, respectively, for the 0.5, 1.0 and 2.5 µM concentrations (Fig. 1B), indicating that FBA-TPQ has selective activity against ovarian cancer cells. In addition, FBA-TPQ inhibited A2780 and OVCAR-3 cells proliferation in a dose-dependent manner (Fig. 1C and 1D), while its anti-proliferative effects were much less evident in the IOSE144 cells treated with the same concentrations of FBA-TPQ (Fig. 1E). In fact, FBA-TPQ significantly inhibited A2780 and OVCAR-3 cells proliferation beginning at the 0.5 µM concentration (Fig. 1C and 1D, p<0.05) while there was no significant inhibition in the IOSE-144 cells at any of the concentrations (up to 1 µM; Fig. 1E, p>0.05). These data suggest that FBA-TPQ can selectively inhibit A2780 and OVCAR-3 ovarian cancer cell growth and proliferation while exerting less potent effects on non-tumorigenic IOSE144 cells.

**Figure 2:**
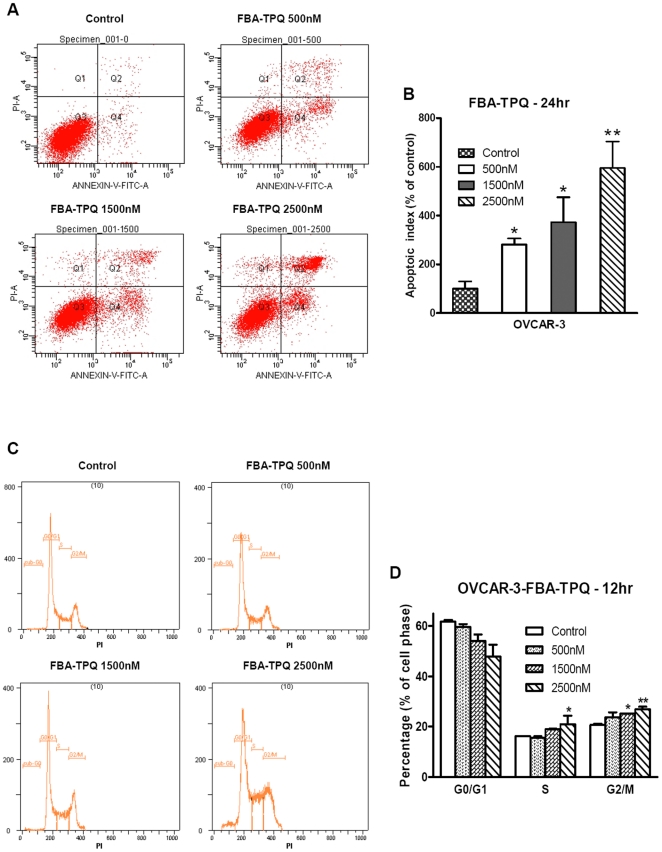
FBA-TPQ induces dose-dependent apoptosis and G2/M cell cycle arrest in OVCAR-3 cells. A, Apoptosis of OVCAR-3 cells treated with serial concentrations of FBA-TPQ for 24 hr; B, Data summary and analysis of the apoptotic index (Q1 reflects necrosis, Q2 reflects late apoptosis, Q3 reflects healthy cell population not affected by apoptosis or necrosis while Q4 reflects early apoptosis). C, Cell cycle evaluation of OVCAR-3 cells treated with serial concentrations of FBA-TPQ for 12 hr; D, Data analysis of cells presented as the percent distribution of a specific phase (*, *p*<0.05 versus the control, **, *p*<0.01 versus the control, respectively). Data are representative of values from at least three independent experiments with similar results.

Since FBA-TPQ dramatically reduced OVCAR-3 cell survival and proliferation, we next chose this more FBA-TPQ-sensitive ovarian cancer cell line to investigate the underlying mechanism responsible for the decrease in cell viability exposed to the compound. First, we evaluated whether FBA-TPQ can induce OVCAR-3 cells' apoptosis. As shown in Fig. 2A and 2B, FBA-TPQ strongly induced apoptosis in a dose-dependent manner during 24 hr exposure. At 0.5 µM concentration, FBA-TPQ increased apoptosis in the OVCAR-3 cells to 260% (of the control cells), while at 1.5 µM concentration, the apoptosis index was increased by nearly 4-fold (Fig. 2B, p<0.05). The pro-apoptotic effects were more profound at the concentration of 2.5 µM (apoptotic index increased to about 600% compared to control) (Fig. 2B, p<0.01). To conclude, our data demonstrated that FBA-TPQ can induce significant apoptosis in the OVCAR-3 cells in a dose-dependent manner.

We also investigated whether FBA-TPQ has any effect on cell cycle progression in OVCAR-3 cells. For this assay, we decreased the FBA-TPQ incubation time to 12 h to to avoid the high apoptosis induction. As illustrated in Fig. 2C and 2D, after a 12-h treatment, FBA-TPQ induced significant increases in the number of cells in the G_2_/M-phase at the 1500 nM (21.6% increase of control, p<0.05) and 2.5 µM (30.0% increase of control, p<0.05) concentrations. In addition, FBA-TPQ treatment also led to a significant increase in the number of cells in the S-phase at the 2.5 µM concentration (Fig. 2D, p<0.05). As summarized in Fig. 2D, FBA-TPQ caused a concomitant, dose-dependent, decrease in the number of cells in the G0/G1-phase (p<0.01). Taken together, our data suggest that the inhibitory effects of FBA-TPQ on cell viability and growth may be associated with its induction of apoptosis and cell cycle arrest.

**Figure 3:**
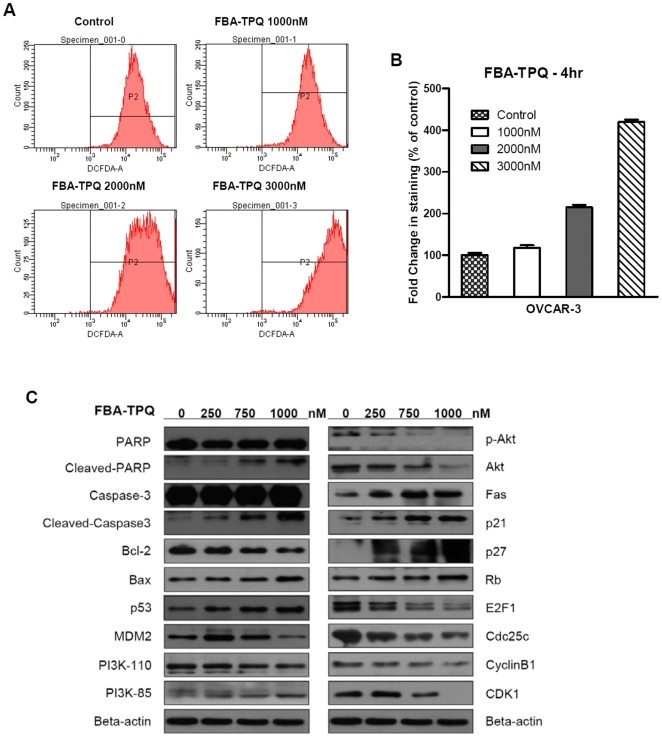
FBA-TPQ induces cellular ROS and regulates the p53-MDM2, and PI3K-Akt mediated pathways. A&B, FBA-TPQ induces dose-dependent ROS stress in OVCAR-3 cells (A, the dose-dependent increase in the ROS as indicated by CM-H_2_DCFDA; B, Data summarized as the percentage compared with the control). C, Western blot analysis of cellular protein expression levels of the related pathway, indicating FBA-TPQ may take effects through the ROS-accompanied, and PI3K-Akt-mediated/p53-MDM2-related cell proliferation, apoptosis and cell cycle progression associated pathway on OVCAR-3 ovarian cancer cell lines after 24 h exposure (at 250,750 and 1000 nM concentrations).

**Figure 4:**
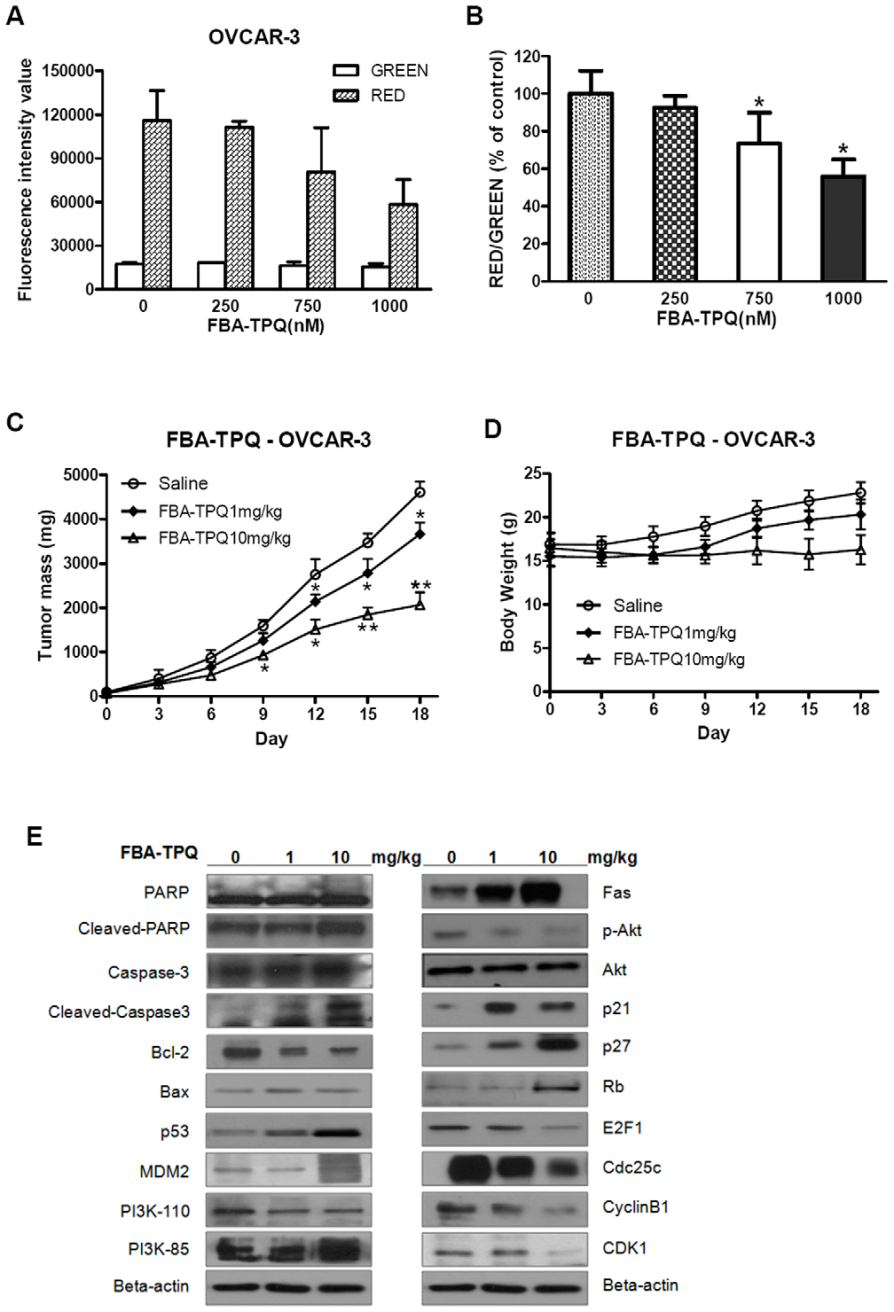
FBA-TPQ causes dissipation of the mitochondrial membrane potential and exerts potent tumor growth inhibition. A and B, Fluorescence intensity (JC-1 produces red fluorescence within the mitochondria as JC-1-aggregates while emits green fluorescence when leaks into the cytoplasm as JC-1-monomers; Fluorescence intensity shift between green and red is proportional to the *ΔΨm* change) values of JC-1 dye at specific excitation wavelengths and the corresponding ratio of Red/Green change (% of the control) after exposure to FBA-TPQ for 24 h (*, *p*<0.05 versus the control). C&D, Inhibition of tumor growth in mice bearing OVCAR-3 xenograft tumors (*, *p*<0.05 versus the control; **, *p*<0.01 versus the control), and the corresponding body weight changes during the treatments (*p*>0.05); E, Western-blot analysis of proteins (of the mice xenograft tumors after FBA-TPQ treatment of 0, 1 or 10 mg/kg dosage) involved in the FBA-TPQ-triggered, and PI3K-Akt mediated/p53-MDM2-related pathway.

In our previous work, we reported that FBA-TPQ may exert DNA damage in breast cancer cells [6]; here, we investigated whether FBA-TPQ can induce cellular ROS stress and cause oxidative damage to the DNA in ovarian cancer cells. In this assay, we decreased the incubation time again to 4 h (to avoid ROS attenuation) while increased the serial concentrations to 0, 1.0, 2.0 and 3.0 µM (to produce quick ROS induction for grabbing the image). As shown in Fig. 3A and 3B, after 4 hrs of incubation, FBA-TPQ induced a significant (p<0.05) dose-dependent increase in cellular ROS production in OVCAR-3 cells (Fig. 3B). Based on our observation that FBA-TPQ could induce apoptosis, we evaluated the mitochondria trans-membrane potential (*ΔΨm*) dissipation as an indicator of mitochondrial membrane disruption and cells undergoing apoptosis. Using the JC-1 dye, we observed that FBA-TPQ treatment resulted in a significant (p<0.05) dose-dependent *ΔΨm* dissipation (dose-dependent reduction of JC-1 aggregates is indicated by decreased ratios of red/green fluorescence intensity) after 24 hrs exposure (Fig. 4A and 4B). Taken together, these results provide evidence that FBA-TPQ induces elevated cellular ROS production and loss of mitochondria membrane potential, initiating the endogenous ROS stress-triggered early cellular apoptosis pathway.

**Figure 5:**
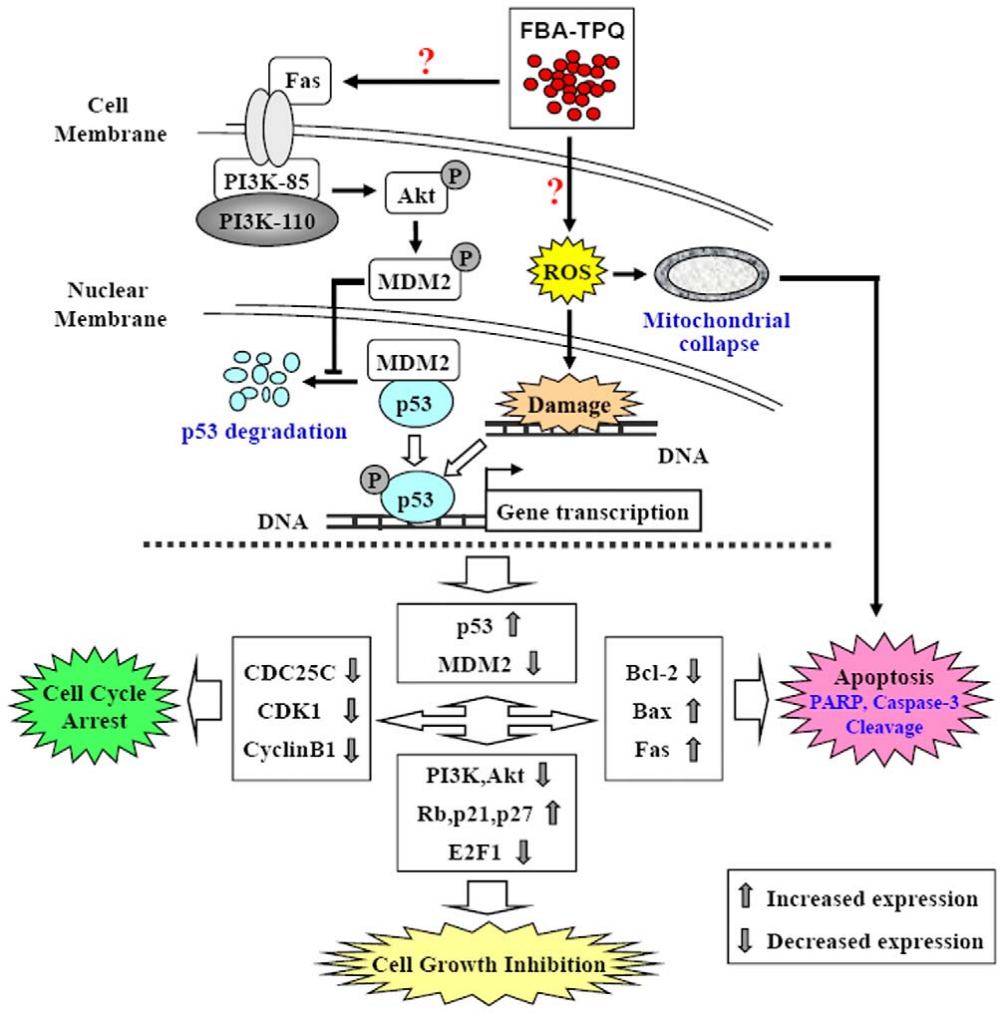
Cartoon of the possible mechanisms of action by which FBA-TPQ excerts its anticancer activities.

To further define the effects of FBA-TPQ on cell apoptosis, cell cycle progression, and proliferation, we investigated the level of expression of a panel of proteins involved in these pathways in OVCAR-3 cells (see Fig. 3C). After a 24-h treatment of FBA-TPQ (with concentrations of 0, 0.25, 0.75 and 1.0 µM), we observed a dose-dependent increase in the expression of cleaved- Poly (ADP-ribose) Polymerase (PARP) and Caspase-3, as well as Bax, with a concomitant dose-dependent decrease in Bcl-2. We further investigated the possible mechanisms responsible for the anti-proliferative and cell cycle regulatory effects of FBA-TPQ. As above, we evaluated the expression level of various proteins associated with proliferation and cell cycle progression, and observed the down-regulation of Cdc25c, CyclinB1 and Cyclin Dependent Kinase (CDK)1, which are responsible for the G2/M phase transition, and found an increase in the expression of Rb, p21 and p27 proteins, as well as a decrease in the E2F1 protein, all of which are involved in cell proliferation (Fig. 3C). Moreover, our data showed that FBA-TPQ can decrease the expression and activation (indicated by phosphorylation) of Akt, as well as its upstream kinase PI3K's catalytic subunit PI3K-110α. We further evaluated the regulatory effects of FBA-TPQ on p53, and found that FBA-TPQ activated p53, with an accompanying down-regulation in MDM2 expression (see Fig. 3C), and increased cleavage of PARP and Caspases-3. These findings are indicative of apoptosis and/or anti-proliferative and cell cycle inhibitory effects. As demonstrated in Fig. 3C, we also observed a dose-dependent up-regulation of Fas, indicating that the death receptor pathway, together with p53, may play a major role in the response to FBA-TPQ and its stimulation of ROS stress. Taken together, we can conclude that FBA-TPQ may exert its *in vitro* effects through the ROS-associated, and PI3K-Akt-mediated/p53-MDM2-related cell proliferation, apoptosis and cell cycle progression associated pathways (Fig. 5).

We established the OVCAR-3 tumor xenograft model to evaluate the *in vivo* anti-tumor activity of FBA-TPQ. The compound was administered 5 days a week i.p. at a dose of 1 mg/kg or 10 mg/kg. Therapeutic effects were evaluated by examining tumor growth. As shown in Fig. 4C, FBA-TPQ exhibits significant (p<0.01) tumor inhibition in a dose-dependent manner. The significant tumor growth inhibitory effects started to be evident on the ninth day of treatment (p<0.05). After 18 days of treatment, FBA-TPQ resulted in 20.5% tumor growth inhibition (compared to the control group) at the lower 1 mg/kg dose, and 69.4% tumor growth inhibition at the higher 10 mg/kg dose (Fig. 4C, p<0.01). Moreover, although the mice receiving the 10 mg/kg dose experienced a slight loss in body weight, there were no significant differences in body weight loss (p>0.05) between the three groups, indicating that FBA-TPQ may have an acceptable safety profile (Fig. 4D).

To validate the *in vitro* findings about the mechanism of action of FBA-TPQ, we further assessed the *in vivo* expression level of the proteins examined in the cultured cells (Fig. 4E). Consistent with the *in vitro* data, the protein expression patterns in the treated xenograft tumor tissue were essentially the same as the cellular observations (see Fig. 3C and Fig. 4E), confirming that the anti-tumor effects of FBA-TPQ are, at least partially mediated through the induction of ROS stress and subsequent mitochondrial collapse, which lead to alterations in cellular cascades that are involved in apoptosis, cell proliferation and cell cycle progression.

## Discussion

The present study is the first to systematically investigate the novel synthetic makaluvamine analog FBA-TPQ's *in vitro* and *in vivo* anti-tumor effects in ovarian cancer cells. We demonstrated that FBA-TPQ exerts its anti-cancer activities on A2780 and OVCAR-3 cells by inhibition of cell growth and proliferation. Further study use the more sensitive OVCAR-3 cell line revealed that it could induce cell apoptosis and cell cycle arrest. Such functional outcomes are achieved through a cascade of reactions within these pathways, including induction of cellular ROS stress, death-receptor activation and mitochondrial collapse, and subsequent regulation of p53-MDM2 and PI3K-Akt associated pathways.

In our previous studies, we showed that FBA-TPQ is the most potent makaluvamine analog with regard to induction of DNA damage, apoptosis, cell cycle arrest and proliferation inhibition in breast cancer cells [6]. However, the possible underlying mechanisms for these anti-tumor activities and the compound's potential application for other cancer types had not been elucidated. The focus of the current study was to determine whether FBA-TPQ could represent a promising candidate for future anti-cancer drug development, and to further elucidate its mechanism(s) of action.

We initially observed that FBA-TPQ exerts significant cytotoxicity and cell proliferation inhibition effects towards A2780 and OVCAR-3 cells. Subsequent evaluation revealed a dose-dependent effect of FBA-TPQ on OVCAR-3 cells apoptosis, and cell cycle progression (G2/M phase cell cycle arrest). These observations were further supported by expression profiling of related proteins in OVCAR-3 cells using Western blotting. In an attempt to explore the mechanism(s) of action underlying the observed biological activities of FBA-TPQ, we found that FBA-TPQ can induce cellular ROS stress and subsequent mitochondrial collapse, providing a potential explanation for the cascade triggering the *in vitro* anticancer effects, including apoptosis. Next, we evaluated the *in vivo* activity of FBA-TPQ in the OVCAR-3 ovarian cancer xenograft model, and showed that FBA-TPQ can again exert potent tumoricidal activity, with up to 69.4% tumor growth inhibition at the 10 mg/kg dose. We systematically (both *in vitro* and *in vivo*) evaluated its underlying mechanism(s) of actions, and our *in vivo* studies confirmed that FBA-TPQ exerts its potent anti-tumor effects mainly by affecting cell proliferation, inducing cell apoptosis and cell cycle arrest.

The cleavage of PARP and procaspase-3 are the well-documented indicators of apoptosis [18], [19]. Members of the Bcl-2 family, such as Bcl-2 and Bax, are known critical regulators responsible for cell survival/apoptosis [20]–[22]. We have demonstrated a dose-dependent cleavage of PARP and procaspase-3, as well as a dose-dependent decrease in the Bcl-2/Bax ratio in FBA-TPQ-treated cells and tumors, providing evidence that FBA-TPQ induces apoptosis. Based on our previous data showing that FBA-TPQ may act as a DNA damaging agent, we evaluated the ROS level in OVCAR-3 cells after FBA-TPQ incubation, as oxidation (together with methylation, depurination and deamination) caused by ROS stress can contribute to DNA damage [23]. Indeed, we observed a dose-dependent increase in the cellular ROS level, as well as a dose-dependent dissipation of the mitochondrial membrane potential. Since the loss of mitochondrial membrane potential is considered an early event in apoptosis [18], [24], we may conclude that FBA-TPQ triggers the observed apoptosis in OVCAR-3 cells by inducing an elevated level of cellular ROS, leading to subsequent mitochondrial membrane collapse [23], [24].

Cells utilize the cell cycle checkpoints to ensure proper cell cycle execution in order to protect dividing cells from potentially fatal DNA damage [25], [26]. Cells may be arrested in the G2/M phase in order to undergo DNA repair or apoptotic cell death [26], [27]. To further investigate the effects of FBA-TPQ on cell cycle arrest as a result of its induction of DNA damage, we evaluated its modulation of the expression of key proteins involved in G2/M signaling, and observed a dose-dependent decrease in the expression of CDC25C and CDK1/Cyclin B1, whose activation are key for G2/M progression [26], [28], [29]. This confirms that FBA-TPQ promotes G2/M arrest of OVCAR-3 cells following ROS-induced damage.

Activation of p53 is a major mechanism of action for many DNA damaging agents [6]. We observed a dose-dependent increase in mutant-p53 expression in OVCAR-3 cells, which is consistent with our previous report that cells respond to FBA-TPQ damage regardless of their p53 status [6]. We also observed down-regulation of MDM2 and a concomitant decrease in E2F1, and increased expression of Rb, p21 and p27 [30], [31], indicating that the activation of the p53-MDM2 feedback loop is likely responsible for the FBA-TPQ treatment-induced increase in the apoptosis and cell cycle arrest and the decrease in cell growth and proliferation in A2780 and OVCAR-3 cells. The Akt signaling pathway plays critical roles in regulating cell survival [32], [33]. The activation of Akt by PI3K (phosphoinositide-3-OH-kinase) kinase can regulate transcriptional factors that are responsible for pro- and anti-apoptotic proteins, as well as growth factors for survival in response to extracellular stimuli or damage [32]. Phosphorylated Akt (p-Akt) is reported to be able to hinder the p53-dependent apoptosis and growth suppression by phosphorylating MDM2 [34]. Our data showed a dose-dependent decrease in p-Akt (and t-Akt) and its upstream activator PI3K (catalytic subunit) and an increase in Fas (death receptor), suggesting that the FBA-TPQ stimulus -suppresses the PI3K-Akt (p-Akt) pathway and phosphorylates MDM2 to regulate the expression of growth factors (E2F1, Rb, p21 and p27, etc.) or apoptotic (PARP, Bcl-2, Bax and caspase-3, etc.) and cell cycle related (CDC25C,CDK1 and Cyclin B1, etc.) proteins, resulting in the compound's sustained anti-cancer effects.

Considering the high mortality rate of ovarian cancer [1]–[4], it is necessity to explore new agents that can act via novel mechanisms of action to improve ovarian cancer therapy. The present study suggests that the most effective makaluvamine analog, FBA-TPQ, may be a novel and promising anti-ovarian cancer agent, in that it can preferentially and potently inhibit ovarian cancer OVCAR-3 cell (but not non-tumorigenic OSE cell) growth. According to our *in vitro* and *in vivo* data, we believe that FBA-TPQ can inhibit ovarian carcinoma growth by triggering the Fas-related/ROS-associated, and PI3K-Akt mediated/p53-MDM2-related increase in cellular apoptosis and cell cycle arrest and the decrease in cell proliferation (Fig. 5). These findings were confirmed at the protein level in *in vitro* and *in vivo*, and were consistent with our microarray data analysis (see Text S1, Fig. S1, Tables S1, S2, S3), that FBA-TPQ exerts its activity mainly through its induction of ROS and DNA damage, regulation of the ‘pathway in cancer’, ‘p53 signaling pathway’ and ‘phosphatidylinositol signaling system’ (e.g., PI3K-Akt), as well as its negatively regulation of CDK (e.g., CDK1 and the related CDC25C and CyclinB1).

In conclusion, our study demonstrated that FBA-TPQ can exert potent cytotoxicity and cell proliferation inhibition effects towards A2780 and OVCAR-3 cells. Except for the induction of apoptosis, it can also induce cell cycle arrest and inhibit OVCAR-3 xenograft tumors' growth through the ROS/death receptor-initiated, and PI3K-Akt mediated/p53-MDM2-related cell proliferation and apoptosis and cell cycle progression-associated pathways (illustrated in Fig. 5). However, the precise mechanism(s) of the compound, and whether it exerts the same effects in other tumors types have yet to be well elucidated. In the future, the present study, together with our previous reported findings [6], will surely improve our understanding about the mechanisms of action of makaluvamine analog and will also provide a basis for their future clinical development of FBA-TPQ as a novel anti-cancer agent.

## Materials and Methods

### Compounds and Reagents

All chemicals were all of analytical grade. FBA-TPQ was a kind gift from Professor Sadanandan Velu (UAB, Birmingham, AL, USA). Propidium iodide (P4170) was bought from Sigma-Aldrich, Inc. (St. Louis, MO, USA). The JC-1 dye (5,5′,6,6′-tetrachloro-1,1′,3, 3′-tetraethylbenzimidazolylcarbocyanine iodide) (T-3186), TRIZOL reagent and CM-H_2_DCFDA [5-(and-6)-chloromethyl-2′, 7′- dichlorodihydrofluorescein diacetate acetyl ester] (C6827) were purchased from Invitrogen-Molecular Probes Co. (City, State, USA). The CellTiter 96® AQ_ueous_ One Solution (G3580) Cell Proliferation Assay (MTS assay) kit was purchased from Promega Co.,Ltd. (Madison, WI). The DC protein assay kit (500-0113) was obtained from Bio-Rad (Hercules, CA, USA), and the ECL plus system was purchased from Amersham Pharmacia Biotech (Buckinghamshire, UK). All cell culture supplies were obtained from Invitrogen-Gibco Co. (City, State, USA). The Anti-human MDM2 antibody was obtained from Calbiochem® of EMD Chemicals Inc. (Darmstadt, Germany), the Caspase-3 (8G10) antibody was purchased from Cell Signaling Technology, Inc. (Danvers, MA, USA), and the anti-human β-actin (AC-74) antibody was obtained from Sigma-Aldrich, Inc. (St. Louis, MO). Anti-human p53 (SC-98), Fas (SC-8009), PI3K110a (SC-7189), PI3K85a (SC-423), AKT (SC-8312), p-AKT (SC-7985-R), PARP (H-250, SC-7150), Bax (SC-493), Bcl-2 (SC-509), Rb (SC-50), p21 (SC-817), p27 (SC-528), E2F1 (SC-193), CDK1 (SC-8395), Cyclin B1 (SC-595), and CDC25C (SC-13138) antibodies, together with all secondary antibodies (anti-mouse, anti-goat and anti-rabbit immunoglobulin G) were purchased from Santa Cruz Biotechnology, Inc. (Santa Cruz, CA, USA).

### Cell Lines and Cell Culture

Human ovarian carcinoma A2780 and OVCAR-3 cells and human ovarian IOSE144 (Immortalized non-tumorigenic Ovarian Surface Epithelial cells) that were originally obtained from the American Type Culture Collection (ATCC, Manassas, VA) were gifts from Dr. Jing Fang (Institute for Nutritional Sciences, Shanghai, China). All cells were cultured in RPMI 1640 medium supplemented with 10% FBS and 1% penicillin/streptomycin according to the ATCC's instructions. FBA-TPQ was dissolved in DMSO then diluted in cell culture media (<0.1%, final incubation concentration).

### Cell Viability Assay

Cell growth and viability were determined by the MTS assay using the CellTiter 96® AQ_ueous_ One Solution Cell Proliferation Assay kit (G3580, Promega). In brief, 4×10^3^ cells (per well) were seeded in 96-well plates and were either treated for 48 h with FBA-TPQ (dissolved in DMSO <0.1%, final concentration) at serial concentrations (0, 100, 500, 750, 1000, 1500 and 2500 nM), or were treated for various times (0, 24, 48 and 72 hr) with FBA-TPQ at concentrations of 0, 500, 750 and 1000 nM. After 24∼72 hrs treatment, 20 µL of MTS solution was added to each well. Plates were incubated for an additional 2∼4 hrs at 37°C, after which the absorbance at 490 nm was recorded using a SpectraMax^190^ microplate reader (Molecular Devices, USA) to calculate the cell survival percentages.

### Detection of Apoptosis

Cell apoptosis was detected via an Annexin V-FITC kit purchased from BioVision, Inc [6], [17]. Briefly, 1×10^6^ cells were seeded in 6-cm dishes and treated with the test compound (FBA-TPQ) at 0.5, 1.5 and 2.5 µM for 24 hr prior to analysis. The floating and trypsinized adherent cells were collected and prepared for detection according to the manufacturer's instructions. Samples were analyzed with a FACSAria™ flow cytometer (Becton Dickinson, USA) after incubation in the dark at room temperature for 5 min. Cells positive for early apoptosis (Annexin V-FITC stained only, see Q_4_ in Figure 2A) and for late apoptosis (Annexin V-FITC and PI stained, see Q_2_ in Figure 2A) were combined.

### Cell Cycle Analysis

Propidium iodide (PI) staining was performed to determine the effects of FBA-TPQ on the cell cycle [6]. In brief, 6×10^5^ cells that were seeded in 6-cm dishes were exposed to FBA-TPQ (at 0, 0.5, 1.5 and 2.5 µM) and incubated for 12 hrs prior to analysis. Cells were trypsinized, washed with PBS and fixed in 1 mL 70% ethanol (700 µL 100% ethanol added to 300 µL PBS) at 4°C overnight, followed by centrifugation (3000 rpm, 5 min), incubation with RNase (100 µg/ml) and staining with propidium iodide (50 µg/ml) for 15 minutes in the dark. The DNA content was analyzed by a Cell Lab Quanta™ SC flow cytometer (Beckman Coulter, USA).

### Measurement of Reactive Oxygen Species (ROS)

The production of ROS was measured using 5-(and-6)-chloromethyl-2′, 7′- dichlorodihydrofluorescein diacetate acetyl ester (CM-H2DCFDA). Briefly, Cells (∼7×10^5^) were seeded in 6-cm dishes and exposed to FBA-TPQ for 4 hours at serial concentrations (0, 1.0, 2.0 and 3.0 µM), after which the cells were harvested and washed with PBS once, then incubated with 5 µM CM-H2DCFDA (in DMSO) for 30 min at 37°C in phenol-red-free RPMI 1640 medium. After the removal of CM-H2DCFDA, cells were washed with PBS again and immediately transferred (in PBS) to a FACSAria™ flow cytometer (Becton Dickinson, USA) for assessment of ROS production.

### Mitochondrial Membrane Potential (ΔΨm) Quantitation

JC-1 dye was used to quantify the effects of FBA-TPQ on the mitochondrial membrane potential, as described previously [17]. OVCAR-3 cells were seeded in 24-well plates (∼4×10^4^ cells per well) and incubated with FBA-TPQ at serial concentrations (0, 250, 750 and 1000 nM) for 24 h. Then the cells were incubated with 10 µg/mL of JC-1 dye in RPMI 1640 at 37°C in the dark for 15 min. After removal of the JC-1 dye, cells were trypsinized and washed with PBS. Aliquots of 100 µL cell suspensions from the different concentrations were transferred to black 96-well plates. The pure red and green fluorescence intensity was measured (for red fluorescence: Ex = 550 nm, Em = 600 nm; for green fluorescence: Ex = 485 nm, Em = 535 nm), via a fluorescence plate reader (Flexstation II 384, Molecular Devices). The ratios of red/green fluorescence intensity (% of control) were calculated. JC-1 staining quantitatively indicated the *ΔΨm* change by shifting the pure fluorescence intensity.

### Western Immunoblotting Analysis

Immunoblotting was accomplished as described previously [6], [17]. For the *in vitro* studies, Cells were cultured and exposed to various concentrations of FBA-TPQ for 24 hr, then were collected and lysed with RIPA buffer (#9806, Cell Signaling). After centrifugation at 13,000 rpm for 15 min at 4°C, the supernatant was removed and kept for analysis. The total protein concentrations were assessed using a Bio-Rad protein assay kit (Hercules, CA). Aliquots containing identical amounts of protein were fractionated by SDS-PAGE, then were transferred to methanol pre-activated-PVDF membranes (Millipore, Bedford, MA). Membranes were blocked for one hour and sequentially incubated with primary (4°C, overnight) and secondary antibody (one hour at room temperature), then bands of the proteins of interest were visualized using the ECL plus system from Amersham Pharmacia Biotech (Buckinghamshire UK).

### Mouse Xenograft Model of Ovarian Cancer and In Vivo Chemotherapy

The tumor xenograft model was established as reported previously [6], [17]. The 5-week-old female athymic nude mice (BALB/c, nu/nu) were purchased from the Shanghai Experimental Animal Center (Shanghai, China). Animal studies were approved by the Institute for Nutritional Sciences (Protocol approval number is 2010-AN-2, with the Protocol title “In vivo and in vitro anticancer activity evaluation and molecular mechanism study of synthetic Makaluvamine Analog for ovarian cancer therapy” and Protocol expiration date “March 1st, 2013”). Briefly, OVCAR-3 cells were harvested and resuspended in serum-free RPMI 1640 medium containing 20% (v/v) Matrigel (BD Biosciences, Bedford, MA). Aliquots of cells (∼5×10^6^ cells/0.15 mL) were injected subcutaneously into the left inguinal area of the mice. The tumor growth and body weight of the mice were monitored every other day. Tumor mass (weight in g) was determined by the formula 1/2*a*×*b* ^2^ where “*a*” is the long diameter and “*b*” is the short diameter (in cm). Mice bearing palpable tumors (about one week after tumor cell inoculation) were randomly divided into control and treatment groups (5 mice/group). FBA-TPQ was dissolved in Cremophor EL∶Ethanol∶Saline (5∶5∶90, v/v/v) and administered (via i.p. injection) at doses of 1 and 10 mg/kg (5 days/week for two and half weeks). The control group received saline only. Mice were sacrificed with CO_2_ gas on Day 18; tumors were carefully excised, and homogenized with RIPA buffer (100 mg tumor tissue/1 ml RIPA) in preparation for immunoblotting analysis as described above.

### Statistical Analysis

All of the values in the present study are reported as the means ± SD from at least three independent experiments. One-way ANOVA was used to test the statistical differences for single group analysis, followed by Tukey's multiple comparisons. Two-way ANOVA was used for grouped analysis of statistical differences, followed by Bonferroni post-tests.

## Supporting Information

Figure S1 **Microarray data support the possible molecular mode of action of FBA-TPQ.** A, GO category of the up-regulated genes based on biological processes for differentially expressed genes (p-value<0.05 and FDR<0.05 were used as a threshold to select significant GO categories; LgP is the logarithm of the p-value); B, KEGG pathway analysis of significant pathways for differentially up-regulated genes (p-value<0.05 and FDR<0.05 were used as a threshold to select significant KEGG pathways; LgP is the logarithm of the p-value). C, Pathway-net indicating the interactions of KEGG pathways (the arrow shows how the signals flow from the source pathway to the target pathway; the red and blue balls represent the up-regulated or down-regulated pathways; the red ball with a blue ring indicates a critical pathway that is both up-regulated and down-regulated).
(TIF)

Table S1 **Significant GOs up-regulated by FBA-TPQ.**
(DOC)

Table S2 **Significant pathways up-regulated by FBA-TPQ.**
(DOC)

Table S3 **The interaction net of significant pathways.**
(DOC)

Text S1 **Supplemental data.**
(DOC)
