# Supplementary figures and images for: Experimental Therapy of Ovarian Cancer with Synthetic Makaluvamine Analog: In Vitro and In Vivo Anticancer Activity and Molecular Mechanisms of Action
Source: PLoS One. 2011 Jun 6;6(6):e20729. doi: 10.1371/journal.pone.0020729 (PMC3108973; doi:10.1371/journal.pone.0020729)

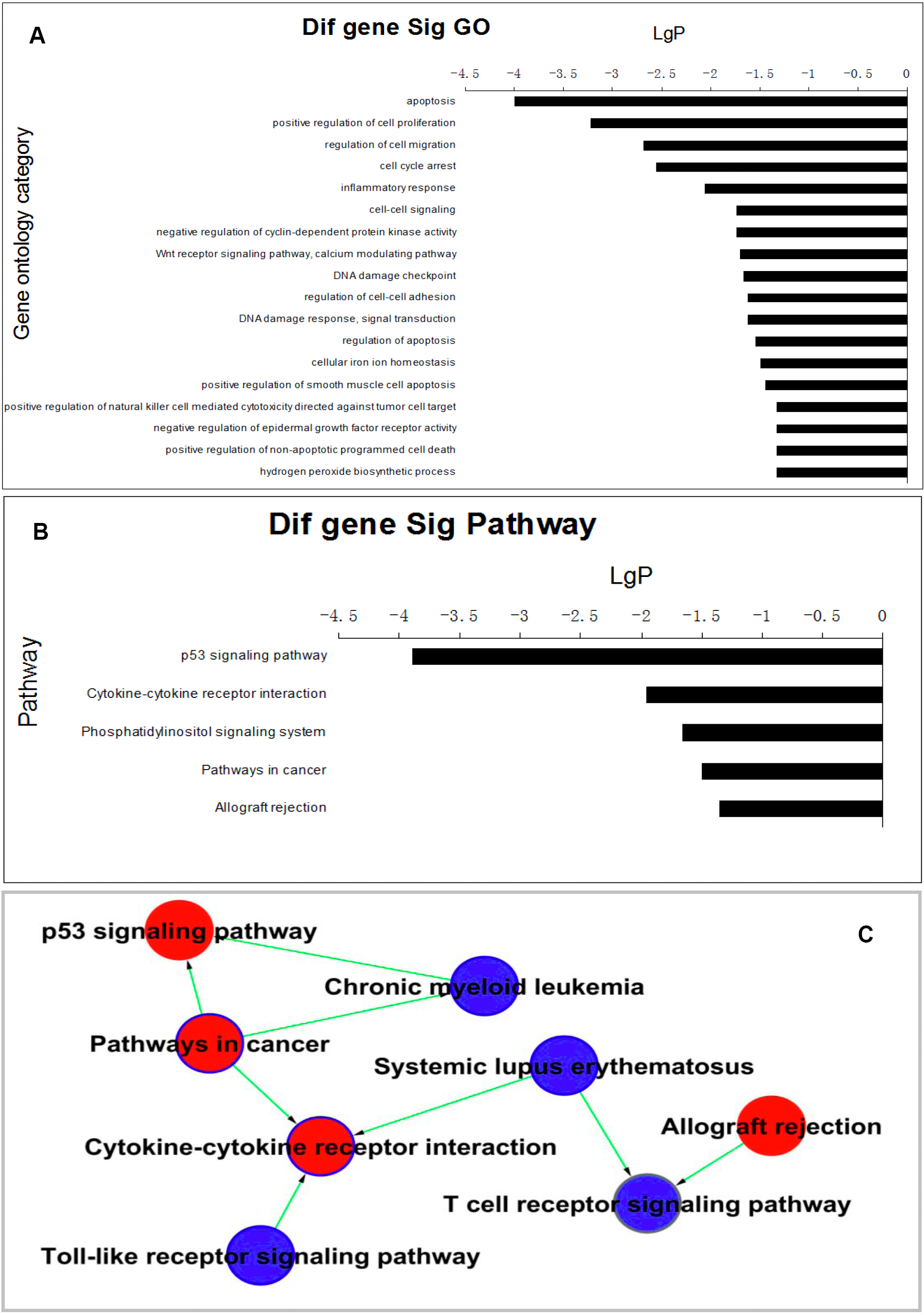

Supplement: Figure S1 — Microarray data support the possible molecular mode of action of FBA-TPQ. A, GO category of the up-regulated genes based on biological processes for differentially expressed genes (p-value<0.05 and FDR<0.05 were used as a threshold to select significant GO categories; LgP is the logarithm of the p-value); B, KEGG pathway analysis of significant pathways for differentially up-regulated genes (p-value<0.05 and FDR<0.05 were used as a threshold to select significant KEGG pathways; LgP is the logarithm of the p-value). C, Pathway-net indicating the interactions of KEGG pathways (the arrow shows how the signals flow from the source pathway to the target pathway; the red and blue balls represent the up-regulated or down-regulated pathways; the red ball with a blue ring indicates a critical pathway that is both up-regulated and down-regulated). (TIF) [file pone.0020729.s001.tif]
