# Supplementary material for: Experimental Therapy of Ovarian Cancer with Synthetic Makaluvamine Analog: In Vitro and In Vivo Anticancer Activity and Molecular Mechanisms of Action
Source: PLoS One. 2011 Jun 6;6(6):e20729. doi: 10.1371/journal.pone.0020729 (PMC3108973; doi:10.1371/journal.pone.0020729)
Supplement: Table S1 — Significant GOs up-regulated by FBA-TPQ. (DOC) [file pone.0020729.s002.doc]

**Table S1. Significant GOs up-regulated by FBA-TPQ**

| **GO name** | **p-value** | **FDR** | **Enrichment** | **Gene symbol** |
| --- | --- | --- | --- | --- |
| Apoptosis | 1.02E-04 | 2.07E-03 | 3.36 | *TP53I3,DRAM,ITGB2,BIK,INPP5D,FAS,F2,PRAMEF2,EGLN3,NTN1,ARC,CXCR4,PHLDA2,KIAA1244,CYFIP2* |
| Positive regulation of cell proliferation | 5.95E-04 | 3.17E-03 | 4.80 | *SHH,CSF1,FOSL1,ADM,NTN1,GDF15,ARC,EGR4* |
| Regulation of cell migration | 2.10E-03 | 4.54E-03 | 15.00 | *CXCR4,NTN1,SERPINB8* |
| Cell cycle arrest | 2.78E-03 | 5.09E-03 | 6.16 | *BTG4,SESN1,CDKN1A,ARC,IL12A* |
| Inflammatory response | 8.83E-03 | 6.80E-03 | 3.47 | *ITGB2,GRIP2,FOS,CCL20,ALOX5,PTX3CXCR4* |
| Cell-cell signaling | 1.84E-02 | 7.40E-03 | 3.01 | *ITGB2,WNT4,SHH,ADM,CCL20,GDF15* |
| Negative regulation of cyclin-dependent protein kinase activity | 1.85E-02 | 7.40E-03 | 13.75 | *HTN1,CDKN1A* |
| Wnt receptor signaling pathway, calcium modulating pathway | 2.00E-02 | 7.45E-03 | 13.20 | *WNT4,RORA* |
| DNA damage checkpoint | 2.16E-02 | 7.49E-03 | 12.69 | *RRAD,CDS1* |
| Regulation of cell-cell adhesion | 2.42E-02 | 7.55E-03 | 82.48 | *MEGF6* |
| DNA damage response, signal transduction | 2.42E-02 | 7.55E-03 | 82.48 | *CDS1* |
| Regulation of apoptosis | 2.87E-02 | 8.15E-03 | 4.31 | *BIK,TRIM48,FAS,ARC* |
| Cellular iron ion homeostasis | 3.22E-02 | 8.55E-03 | 10.31 | *TFRC,ARC* |
| Positive regulation of smooth muscle cell apoptosis | 3.62E-02 | 8.95E-03 | 54.99 | *IL12A* |
| Positive regulation of natural killer cell mediated cytotoxicity directed against tumor cell target | 4.81E-02 | 1.02E-02 | 41.24 | *IL12A* |
| Negative regulation of epidermal growth factor receptor activity | 4.81E-02 | 1.02E-02 | 41.24 | *IL22RA1* |
| Positive regulation of non-apoptotic programmed cell death | 4.81E-02 | 1.02E-02 | 41.24 | *CDKN1A* |
| Hydrogen peroxide biosynthetic process | 4.81E-02 | 1.02E-02 | 41.24 | *DUOX1* |
