# Supplementary material for: Experimental Therapy of Ovarian Cancer with Synthetic Makaluvamine Analog: In Vitro and In Vivo Anticancer Activity and Molecular Mechanisms of Action
Source: PLoS One. 2011 Jun 6;6(6):e20729. doi: 10.1371/journal.pone.0020729 (PMC3108973; doi:10.1371/journal.pone.0020729)
Supplement: Table S2 — Significant pathways up-regulated by FBA-TPQ. (DOC) [file pone.0020729.s003.doc]

**Table S2. Significant pathways up-regulated by FBA-TPQ**

| **Path name** | **p-value** | **FDR** | **Enrichment** | **Gene name** |
| --- | --- | --- | --- | --- |
| p53 signaling pathway | 1.29E-04 | 7.85E-03 | 11.95 | *TP53I3,CDS1,SESN1,FAS,CDKN1A* |
| Cytokine-cytokine receptor interaction | 1.11E-02 | 2.83E-02 | 3.76 | *FAS,CXCR4,IL12A,CSF1,CCL20,IL22RA1* |
| Phosphatidylinositol signaling system | 2.24E-02 | 3.55E-02 | 6.51 | *CDS1,INPP1,INPP5D* |
| Pathways in cancer | 3.21E-02 | 3.85E-02 | 2.99 | *FAS,FOS,CDKN1A,SHH,WNT4,EGLN3* |
| Allograft rejection | 4.46E-02 | 4.08E-02 | 8.68 | *FAS,IL12A* |
