# Supplementary material for: Experimental Therapy of Ovarian Cancer with Synthetic Makaluvamine Analog: In Vitro and In Vivo Anticancer Activity and Molecular Mechanisms of Action
Source: PLoS One. 2011 Jun 6;6(6):e20729. doi: 10.1371/journal.pone.0020729 (PMC3108973; doi:10.1371/journal.pone.0020729)
Supplement: Table S3 — The interaction net of significant pathways. (DOC) [file pone.0020729.s004.doc]

**Table S3. The interaction net of significant pathways**

| **Source pathway** | **Target pathway** |
| --- | --- |
| Pathways in cancer | Chronic myeloid leukemia |
| Pathways in cancer | Cytokine-cytokine receptor interaction |
| Pathways in cancer | p53 signaling pathway |
| Allograft rejection | T cell receptor signaling pathway |
| Chronic myeloid leukemia | p53 signaling pathway |
| Systemic lupus erythematosus | Cytokine-cytokine receptor interaction |
| Systemic lupus erythematosus | T cell receptor signaling pathway |
| Toll-like receptor signaling pathway | Cytokine-cytokine receptor interaction |
