# Supplementary material for: Experimental Therapy of Ovarian Cancer with Synthetic Makaluvamine Analog: In Vitro and In Vivo Anticancer Activity and Molecular Mechanisms of Action
Source: PLoS One. 2011 Jun 6;6(6):e20729. doi: 10.1371/journal.pone.0020729 (PMC3108973; doi:10.1371/journal.pone.0020729)
Supplement: Text S1 — Supplemental data. (DOC) [file pone.0020729.s005.doc]

**Supplemental Data**

**Experimental therapy of ovarian cancer with synthetic Makaluvamine Analog: *In vitro* and *in vivo* anticancer activity and molecular mechanisms of action**

**Tao Chen^1,†^, Yi Xu^1,†^, He Guo^1^, Yanling Liu^1^, Pingting Hu^1^, Xinying Yang^1^, Xiaoguang Li^1^, Shichao Ge^1^, Sadanandan E. Velu^2^, Dwayaja H. Nadkarni^2^, Wei Wang^3^, Ruiwen Zhang^3^ and Hui Wang^1,*^**

*^1^Key Laboratory of Nutrition and Metabolism, Institute for Nutritional Sciences, Shanghai Institutes for Biological Sciences, Chinese Academy of Sciences, Graduate School of the Chinese Academy of Sciences, Shanghai, P. R. China. ^2^Department of Chemistry, University of Alabama at Birmingham, Birmingham, AL 35294. ^3^Department of Pharmaceutical Sciences, School of Pharmacy, Texas Tech University Health Sciences Center, Amarillo, TX 79106*

**Running title:** Novel Makaluvamine as Anti-Ovarian Cancer Agent

^†^ Authors equally contributed to this work

***Correspondence and requests for reprints:** Hui Wang, MD, PhD, Institute for Nutritional Sciences, Shanghai Institutes for Biological Sciences, Chinese Academy of Sciences, Rm 427, NO.41 Building, 320 YueYang Road, Shanghai, 200031, P. R. China, Tel: +86-21-5492-0941; FAX: +86-21-54920291; E-mail: [huiwang@sibs.ac.cn](mailto:huiwang@sibs.ac.cn)

**Results**

Microarray analysis was performed to evaluate the changes in genes expression patterns between FBA-TPQ-treated and non-treated OVCAR-3 cells. Genes that exhibited a ≥2-fold increase (up-regulated genes) or ≤0.5-fold decrease (down-regulated genes) were chosen for the Gene Ontology (GO) analysis of the main function of these differentially expressed genes. As shown in Fig. S1, FBA-TPQ up-regulated GO groups (GOs) were mainly associated with apoptosis, regulation of cell proliferation, cell cycle arrest, negative regulation of CDKs (cyclin-dependent protein kinases), DNA damage checkpoint, and the hydrogen peroxide biosynthetic process (Fig.S1A and Table S1). This is consistent with our *in vitro* and *in vivo* data which showed that FBA-TPQ induces ROS stress and the DNA damage response, apoptosis, inhibits proliferation and induces G2/M phase arrest through negative regulation of CDK1 and the related CDC25C and CyclinB1.

To further define the signaling pathways that are significantly regulated by FBA-TPQ, the KEGG pathway analysis was performed. The results demonstrated that the p53 signaling pathway and the phosphatidylinositol signaling system were significantly up-regulated (Fig. S1B and Table S2). Moreover, a pathway-net was built to show the relationship between the significant pathways affected by FBA-TPA (Fig. S1C and Table S3). As shown, the ‘pathway in cancer’ is the source pathway, and plays a central role in the signaling network involved in the activity of FBA-TPQ. The ‘p53 signaling pathway’ and ‘cytokine-cytokine receptor interaction pathway’ are two of the other major targeted pathways that are responsible for molecular messages flowing from the ‘pathway in cancer’ to the ‘p53’ and ‘cytokine-cytokine receptor interaction’ associated pathways following FBA-TPQ treatment. Altogether, the microarray data confirms that FBA-TPQ exerts its activity mainly through the induction of ROS and DNA damage, regulating the ‘pathway in cancer’, ‘p53 signaling pathway’ and ‘phosphatidylinositol signaling system’(PI3K-Akt), and by negatively regulating CDK to inhibit proliferation and induce cell cycle arrest and apoptosis.

**Material and methods**

**Microarray**

The microarrays were performed as described [1]. Briefly, OVCAR-3 cells were seeded in 10 cm dishes and treated with 1000nM FBA-TPQ or solvent (DMSO; <0.1% of the final incubation concentration) for 24 hours. Total RNA was extracted and purified using the TRIZOL reagent and the RNeasy® Mini Kit (Qiagen, Hilden, Germany), respectively. Purified RNA was labeled with a RNA Fluorescent Linear Amplification Kit (Agilent Technologies, Palo Alto, CA). The cy3 labeled sample and cy5 labeled control were fragmented by incubation at 60°C for 30 minutes in fragmentation buffer (Agilent Technologies). The fragmentation reaction was stopped by adding an equal volume of Gene Expression Hybridization Buffer (Agilent Technologies). The fragmented target was hybridized to 4*44K whole human genome microarrays (Agilent Technologies) at 60°C for 17 hours in a hybridization oven (Robbins Scientific, Sunnyvale, CA). Following hybridization, the slides were washed with the Gene Expression Wash Buffer Kit (Agilent Technologies) and scanned with an Agilent microarray scanner (Agilent Technologies). The Feature Extraction Software (Agilent Technologies) was used for feature data extraction, and GeneSpring 10.0.was used to perform data analysis. The LOWESS method was used for normalizing the Agilent microarrays.

**GO Category and Pathway Analysis**

GO analysis was applied to analyze the main function of the differential genes expression according to the Gene Ontology, which is the key functional classification of the NCBI [2-4]. Generally, Fisher’s exact test and the test were used to classify the GO category, and the false discovery rate (FDR) [3] was calculated to correct the p-value. The FDR correlated with the error, with a smaller FDR correlating with a smaller error in judging the p-value. The FDR was defined as, where refers to the number of Fisher’s test P-values less thantest P-values. We computed P-values for the GOs of all of the differential genes. Similarly, pathway analysis was used to uncover the significant pathway related to the differential genes, according to KEGG, Biocarta and Reatome. We still used the Fisher’s exact test and test to select the significant pathway, and the threshold of significance was defined by the P-value and FDR. The enrichment Re was calculated as above [5-7]. The Path-Net was the interaction net of the significant pathways of the differentially-expressed genes, and was built according to the interaction among pathways of the KEGG database to find the interaction among the significant pathways directly and systemically. This net can be used to summarize the pathway interaction of differentially expressed genes under disease and normal states, and can be used to determine the reason why a certain pathway was activated [7].

**References**

1. Liao J, Cui C, Chen S, et al. (2009) Generation of induced pluripotent stem cell lines from adult rat cells. Cell Stem Cell 4:11-5.
2. The Gene Ontology (GO) project in 2006 (2006). Nucleic acids research 34:D322-6.
3. Ashburner M, Ball C A, Blake J A, et al. (2000) Gene ontology: tool for the unification of biology. The Gene Ontology Consortium. Nature genetics 25:25-9.
4. Dupuy D, Bertin N, Hidalgo C A, et al. (2007) Genome-scale analysis of in vivo spatiotemporal promoter activity in Caenorhabditis elegans. Nature biotechnology 25:663-8.
5. Kanehisa M, Goto S, Kawashima S, Okuno Y, Hattori M (2004) The KEGG resource for deciphering the genome. Nucleic acids research 32:D277-80.
6. Yi M, Horton J D, Cohen J C, Hobbs H H, Stephens R M (2006) WholePathwayScope: a comprehensive pathway-based analysis tool for high-throughput data. BMC bioinformatics 7:30.
7. Draghici S, Khatri P, Tarca a L, et al. (2007) A systems biology approach for pathway level analysis. Genome research 17:1537-45.

**Table S1. Significant GOs up-regulated by FBA-TPQ**

| **GO name** | **p-value** | **FDR** | **Enrichment** | **Gene symbol** |
| --- | --- | --- | --- | --- |
| Apoptosis | 1.02E-04 | 2.07E-03 | 3.36 | *TP53I3,DRAM,ITGB2,BIK,INPP5D,FAS,F2,PRAMEF2,EGLN3,NTN1,ARC,CXCR4,PHLDA2,KIAA1244,CYFIP2* |
| Positive regulation of cell proliferation | 5.95E-04 | 3.17E-03 | 4.80 | *SHH,CSF1,FOSL1,ADM,NTN1,GDF15,ARC,EGR4* |
| Regulation of cell migration | 2.10E-03 | 4.54E-03 | 15.00 | *CXCR4,NTN1,SERPINB8* |
| Cell cycle arrest | 2.78E-03 | 5.09E-03 | 6.16 | *BTG4,SESN1,CDKN1A,ARC,IL12A* |
| Inflammatory response | 8.83E-03 | 6.80E-03 | 3.47 | *ITGB2,GRIP2,FOS,CCL20,ALOX5,PTX3CXCR4* |
| Cell-cell signaling | 1.84E-02 | 7.40E-03 | 3.01 | *ITGB2,WNT4,SHH,ADM,CCL20,GDF15* |
| Negative regulation of cyclin-dependent protein kinase activity | 1.85E-02 | 7.40E-03 | 13.75 | *HTN1,CDKN1A* |
| Wnt receptor signaling pathway, calcium modulating pathway | 2.00E-02 | 7.45E-03 | 13.20 | *WNT4,RORA* |
| DNA damage checkpoint | 2.16E-02 | 7.49E-03 | 12.69 | *RRAD,CDS1* |
| Regulation of cell-cell adhesion | 2.42E-02 | 7.55E-03 | 82.48 | *MEGF6* |
| DNA damage response, signal transduction | 2.42E-02 | 7.55E-03 | 82.48 | *CDS1* |
| Regulation of apoptosis | 2.87E-02 | 8.15E-03 | 4.31 | *BIK,TRIM48,FAS,ARC* |
| Cellular iron ion homeostasis | 3.22E-02 | 8.55E-03 | 10.31 | *TFRC,ARC* |
| Positive regulation of smooth muscle cell apoptosis | 3.62E-02 | 8.95E-03 | 54.99 | *IL12A* |
| Positive regulation of natural killer cell mediated cytotoxicity directed against tumor cell target | 4.81E-02 | 1.02E-02 | 41.24 | *IL12A* |
| Negative regulation of epidermal growth factor receptor activity | 4.81E-02 | 1.02E-02 | 41.24 | *IL22RA1* |
| Positive regulation of non-apoptotic programmed cell death | 4.81E-02 | 1.02E-02 | 41.24 | *CDKN1A* |
| Hydrogen peroxide biosynthetic process | 4.81E-02 | 1.02E-02 | 41.24 | *DUOX1* |

**Table S2. Significant pathways up-regulated by FBA-TPQ**

| **Path name** | **p-value** | **FDR** | **Enrichment** | **Gene name** |
| --- | --- | --- | --- | --- |
| p53 signaling pathway | 1.29E-04 | 7.85E-03 | 11.95 | *TP53I3,CDS1,SESN1,FAS,CDKN1A* |
| Cytokine-cytokine receptor interaction | 1.11E-02 | 2.83E-02 | 3.76 | *FAS,CXCR4,IL12A,CSF1,CCL20,IL22RA1* |
| Phosphatidylinositol signaling system | 2.24E-02 | 3.55E-02 | 6.51 | *CDS1,INPP1,INPP5D* |
| Pathways in cancer | 3.21E-02 | 3.85E-02 | 2.99 | *FAS,FOS,CDKN1A,SHH,WNT4,EGLN3* |
| Allograft rejection | 4.46E-02 | 4.08E-02 | 8.68 | *FAS,IL12A* |

**Table S3. The interaction net of significant pathways**

| **Source pathway** | **Target pathway** |
| --- | --- |
| Pathways in cancer | Chronic myeloid leukemia |
| Pathways in cancer | Cytokine-cytokine receptor interaction |
| Pathways in cancer | p53 signaling pathway |
| Allograft rejection | T cell receptor signaling pathway |
| Chronic myeloid leukemia | p53 signaling pathway |
| Systemic lupus erythematosus | Cytokine-cytokine receptor interaction |
| Systemic lupus erythematosus | T cell receptor signaling pathway |
| Toll-like receptor signaling pathway | Cytokine-cytokine receptor interaction |
